# Surgical correction of aortic coarctation with an aberrant right subclavian artery in a 5-month-old infant: a case report

**DOI:** 10.1093/jscr/rjag352

**Published:** 2026-08-11

**Authors:** Mohammad Younes, Ali Deeb

**Affiliations:** Cardiac Surgery Department, Faculty of Medicine, Damascus University, Damascus, Syria; Cardiac Surgery Department, Faculty of Medicine, Damascus University, Damascus, Syria

**Keywords:** aortic coarctation, aberrant right subclavian artery, arteria lusoria, infant, cardiac surgery, case report

## Abstract

A 5-month-old infant with critical juxtaductal coarctation (CoA) and an aberrant right subclavian artery (ARSA) underwent successful single-stage repair. Preoperative computed tomography angiography (CTA) confirmed the ARSA originating distal to the coarctation shelf, a critical finding for surgical planning. Through a left thoracotomy, the ARSA was ligated and divided to prevent vascular steal and allow tension-free mobilization. After confirming adequate collateral perfusion to the right arm, an extended end-to-end anastomosis was performed to resect the coarctation. The postoperative course was uncomplicated, with no residual gradient or limb ischemia. This case underscores the importance of preoperative CTA for detecting arch anomalies like ARSA in CoA patients. A tailored approach with initial ARSA division facilitates a successful extended end-to-end repair, ensuring excellent hemodynamic outcomes and preserving upper limb viability.

## Introduction

Coarctation of the aorta (CoA) accounts for 5%–8% of congenital heart anomalies [1]. While isolated CoA repair is standardized, associated arch vessel anomalies significantly increase surgical complexity. Aberrant right subclavian artery (ARSA), occurring in 0.5%–2% of the general population and up to 3%–5% of CoA patients, is the most frequent such anomaly [2]. When ARSA originates distal to the coarctation shelf, it acts as a tether, complicating mobilization and raising risks of residual obstruction or limb ischemia.

Delayed presentation of critical CoA beyond the neonatal period can lead to severe complications, including dilated cardiomyopathy and, rarely, bronchial compression from mass effect. Managing an infant presenting with this triad—critical CoA, ARSA, and severe heart failure—poses distinct surgical and perioperative challenges [3].

This case report details the successful management of a 5-month-old infant with this complex presentation. We highlight the pivotal role of advanced imaging in diagnosis, describe a tailored surgical strategy for ARSA management, and document remarkable recovery of ventricular function following relief of obstruction.

## Case report

A 5-month-old female infant, weighing 5 kg, born at full term with an unremarkable history, was referred to our center for progressive signs of heart failure. Initially asymptomatic at birth, she developed symptoms around 1 month of age, including worsening tachypnea, diaphoresis during feeds, and faltering growth, consistent with delayed presentation of critical left-sided obstruction [4].

Upon admission, vital signs revealed significant upper-to-lower extremity blood pressure discrepancy (120/70 mmHg in the left arm vs. 80/45 mmHg in the left leg) with weak and delayed femoral pulses. The infant was tachypneic (55 breaths/min) but normoxic. Cardiac auscultation revealed a gallop rhythm. The liver edge was palpable 3 cm below the costal margin, indicating congestive heart failure.

Transthoracic echocardiography confirmed critical juxtaductal coarctation with a prominent posterior shelf. The most striking finding was severe left ventricular dilation and systolic dysfunction, with an ejection fraction of 38% and significant wall thinning—a phenotype of secondary cardiomyopathy due to chronic pressure overload [5]. The study also suggested an aberrant origin of the right subclavian artery.

**Figure 1:**
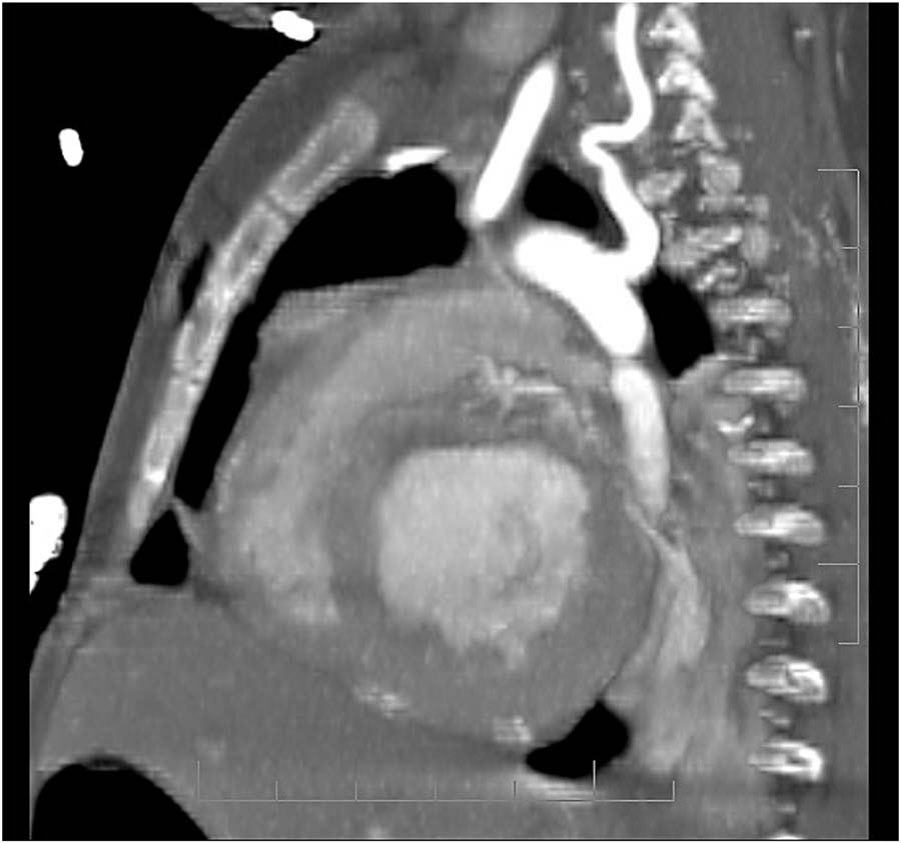
Three-dimensional CT reconstruction showing the aortic arch with coarctation (narrowing) and the ARSA originating from the descending aorta distal to the coarctation site.

To definitively delineate the complex anatomy for surgical planning, computed tomography angiography with 3D reconstruction was performed. This confirmed an ARSA originating from the proximal descending aorta, just distal to the coarctation shelf (Fig. 1). Additionally, compression of the left main bronchus by the dilated aortic arch was noted, explaining the respiratory component of her heart failure [6].

**Figure 2:**
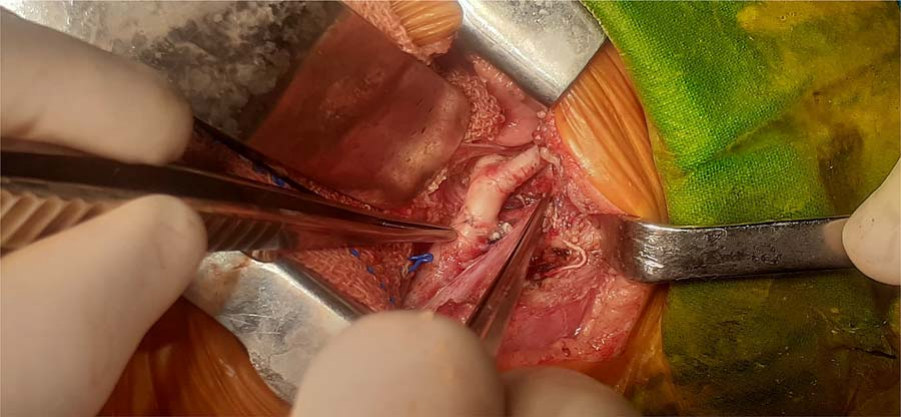
Surgical illustration showing the native anatomy (coarctation and ARSA) and post-repair anatomy after ARSA ligation and extended end-to-end aortic anastomosis.

Given the diagnosis of critical coarctation with heart failure, urgent surgical correction was undertaken via left posterolateral thoracotomy without cardiopulmonary bypass. The ARSA was meticulously dissected, ligated, and divided. Following ligation, right upper limb perfusion remained satisfactory with no compromise in pulse or pressure. This step was essential to achieve adequate mobilization of the descending aorta and permit radical, tension-free resection of all coarcted tissue. Following extensive mobilization, the coarctation segment and hypoplastic isthmus were completely resected. A primary extended end-to-end anastomosis was constructed between the undersurface of the aortic arch and the descending aorta [4]. Aortic cross-clamp time was 17 minutes (Fig. 2).

The immediate postoperative period focused on managing pre-existing cardiomyopathy. Perfusion to the right upper extremity remained excellent with palpable radial pulses. Due to severely depressed myocardial function, the infant required non-invasive ventilatory support for 48 hours before successful extubation. She was discharged home on postoperative day 5 on anti-failure medication.

Follow-up at 6 months demonstrated significant recovery of left ventricular function (EF improved to 55%) [7]. She exhibited excellent growth, complete resolution of heart failure symptoms, and normalized limb blood pressures. Surveillance imaging confirmed resolution of the bronchial compression.

## Discussion

This case presents a complex clinical scenario involving critical aortic coarctation, an ARSA with associated bronchial compression, and severe secondary dilated cardiomyopathy. ARSA occurs in 3%–5% of coarctation cases and poses significant surgical considerations [2]. Originating from the proximal descending aorta distal to the coarctation, the aberrant artery acts as a tether, limiting mobilization and risking residual obstruction. Our decision to ligate and divide the ARSA, rather than perform reimplantation, was guided by clinical priorities. Following ligation, right upper limb perfusion remained satisfactory. In a symptomatic infant with heart failure, the primary goal is definitive coarctation resection to relieve afterload. Reimplantation is technically demanding, prolongs cross-clamp time, and may compromise anastomotic integrity. Ligation is safe when robust collaterals exist, a strategy supported by recent literature [2]. The absence of postoperative ischemia validated this approach.

The severe left ventricular dysfunction (ejection fraction 38%) reflects the profound impact of chronic pressure overload [5]. Importantly, such ‘cardiomyopathic’ changes are often reversible after afterload correction. Our patient’s recovery of LV function to 55% over 6 months underscores that even severe dysfunction should not delay surgery, as the underlying pathology is mechanical rather than intrinsic myocardial disease [8]. Additionally, CTA-documented bronchial compression explained the respiratory component of her heart failure [6]. Postoperative improvement confirmed that addressing aortic pathology resolves secondary airway compromise without separate intervention.

We performed an extended end-to-end anastomosis without cardiopulmonary bypass, with a 17-minute cross-clamp time. This technique offers growth potential and low recurrence risk [7]. Avoiding bypass minimized systemic inflammation and coagulopathy risks.

While 6-month follow-up shows excellent results, long-term surveillance for re-coarctation, aneurysm, or residual dysfunction remains essential. Nonetheless, this case demonstrates that meticulous preoperative planning and a tailored surgical strategy achieve outstanding outcomes.

## Conclusion

This case demonstrates that complex infantile coarctation with ARSA and severe cardiomyopathy can be successfully managed through comprehensive preoperative imaging, a flexible surgical strategy prioritizing definitive arch repair with ARSA ligation, and anticipatory postoperative care [3]. Even profound secondary cardiomyopathy is reversible with timely relief of aortic obstruction, highlighting the importance of systematic evaluation for arch anomalies.

## References

[1] Hoffman JI, Kaplan S. The incidence of congenital heart disease. J Am Coll Cardiol 2002;39:1890–900. 10.1016/s0735-1097(02)01886-7 12084585

[2] Hjortdal VE, Sørensen K, Hansen OK. Implications of anomalous right subclavian artery in the repair of neonatal aortic coarctation. Ann Thorac Surg 2003;76:572–5. 10.1016/s0003-4975(03)00431-4 12902106

[3] Juraszek AL, Guleserian KJ. Preoperative imaging of aortic arch anomalies in children: when is CTA or MRI indicated? Cardiol Young 2016;26:1467–74.

[4] Cobanoglu A, Thyagarajan GK. The extended end-to-end anastomosis for repair of coarctation in infants and children: a 25-year experience. Ann Pediatr Cardiol 2017;10:244–9.

[5] Hwang MS, Chu JJ, Su WJ et al. Dilated cardiomyopathy: an unusual presentation of aortic coarctation in an infant. Cardiology 2006;106:63–6. 10.1159/000092600 16612070

[6] Baird CW, Myers PO, del Nido PJ. Management of associated airway anomalies in patients with congenital heart disease. Semin Thorac Cardiovasc Surg Pediatr Card Surg Annu 2018;21:41–6.

[7] Wright GE, Nowak CA, Goldberg CS et al. Extended resection and end-to-end anastomosis for aortic coarctation in infants: results of a tailored surgical approach. Ann Thorac Surg 2005;80:1453–9. 10.1016/j.athoracsur.2005.04.002 16181886

[8] Lam YY, Kaya MG, Li W et al. Left ventricular remodeling and functional recovery after successful coarctation repair in infants. Int J Cardiol 2019;274:88–93.

